# Investigation of the relationship between intradialytic hypotension during hemodialysis and serum syndecan-1 concentration

**DOI:** 10.1038/s41598-023-44094-7

**Published:** 2023-10-05

**Authors:** Hideaki Oiwa, Hideshi Okada, Keiko Suzuki, Kazuyuki Sumi, Shozo Yoshida, Kodai Suzuki, Takuma Ishihara, Hiroki Kitagaki, Kaori Kimura, Yoshihito Naito, Naokazu Chiba, Ayumi Kuroda, Akihiro Uchida, Hirotsugu Fukuda, Yuki Kawasaki, Toru Minamiyama, Ayane Nishio, Takuto Shimada, Ryo Kamidani, Tomotaka Miura, Ryota Tochibora, Saori Yamamoto, Yujiro Kinomura, Yuichiro Kitagawa, Tetsuya Fukuta, Takahito Miyake, Takahiro Yoshida, Akio Suzuki, Nobuyuki Tetsuka, Hiroyuki Tomita, Takahide Nawa, Shinji Ogura

**Affiliations:** 1 Department of Emergency and Disaster Medicine, Gifu University Graduate School of Medicine, 1-1 Yanagido, Gifu, 501-1194 Japan; 2 Center for One Medicine Innovative Translational Research, Gifu University Institute for Advanced Study, Gifu, Japan; 3 Department of Infection Control, Gifu University Graduate School of Medicine, Gifu, Japan; 4 Department of Pharmacy, Gifu University Hospital, Gifu, Japan; 5 Abuse Prevention Emergency Medicine, Gifu University Graduate School of Medicine, Gifu, Japan; 6 Innovative and Clinical Research Promotion Center, Gifu University Hospital, Gifu, Japan; 7 Gifu Seiryu Hospital, Gifu, Japan; 8 Department of Tumor Pathology, Gifu University Graduate School of Medicine, Gifu, Japan

**Keywords:** Medical research, Biomarkers

## Abstract

Intradialytic hypotension and arrhythmias are complications of hemodialysis. They are associated with decreased intravascular volume due to reduced ultrafiltration volume, cardiac function, and arterial tone. The vascular endothelial glycocalyx, which exists on the surface of healthy vascular endothelial cells and maintains vascular permeability, has been suggested to be impaired by hemodialysis. This single-center retrospective study evaluated the association between syndecan-1, an endothelial glycocalyx dysfunction marker, and complications of hemodialysis. We enrolled 92 patients who underwent outpatient hemodialysis at Gifu Seiryu Hospital from April to July 2022 (346 hemodialysis sessions). The median duration and time of hemodialysis were 40 months and 4.1 h, respectively. Median serum syndecan-1 levels were 67.7 ng/mL before and 98.3 ng/mL after hemodialysis. Hemodialysis complications were noted in 68 sessions, all of which were hypotension. No correlation between pre-hemodialysis syndecan-1 levels and the incidence of complications was observed. However, a positive correlation between the amount of change in syndecan-1 levels before and after hemodialysis and the incidence of hemodialysis complications was noted. Conversely, syndecan-1 levels did not correlate with brain or atrial natriuretic peptides, suggesting that impairment of the vascular endothelial glycocalyx may be a possible cause of intradialytic hypotension and may be useful in preventing intradialytic hypotension.

## Introduction

For patients with end-stage renal failure, hemodialysis (HD) plays an important role in replacing renal function. Despite recent advances in HD technology, HD complications, such as intradialytic hypotension and arrhythmia, occur in clinical practice. This is due to the higher average age of HD patients and the increasing prevalence of complications such as diabetes and heart failure^1,2^. HD complications relate to parameters such as ultrafiltration rate, cardiac function, arteriolar tone, and electrolyte disturbance due to diffusion.

The vascular endothelial glycocalyx is composed of glycoproteins and polysaccharides. It is present on the surface of healthy vascular endothelial cells and plays a role in microcirculatory homeostasis, including the regulation of osmotic pressure and vascular permeability^3,4^. A recent study suggests that HD itself impairs the vascular endothelial glycocalyx and that the greater the amount of water removed per unit time, the more the vascular endothelial glycocalyx is impaired^5^. Disruption of the vascular endothelial glycocalyx may increase vascular permeability, resulting in decreased intravascular volume, possibly leading to intradialytic hypotension and increased susceptibility to arrhythmias, including tachycardia.

Syndecan-1 is a core protein of heparan sulfate proteoglycans. It is one of the components of the endothelial glycocalyx. When the endothelial glycocalyx is injured, syndecan-1 is released from the endothelium, causing an increase in its concentration in circulation^6^. In addition, serum syndecan-1 has been used as an endothelial injury marker in recent clinical studies in critically ill patients^7–9^.

We hypothesized that complications such as hypotension and arrhythmia during hemodialysis may involve a decrease in intravascular volume caused by damage to vascular endothelial glycocalyx, and we quantified vascular endothelial glycocalyx injury using syndecan-1 concentration measurement caused by HD and examined whether the degree of damage was related to events during HD.

## Results

### Characteristics of patients

In total, 92 patients (24 females and 68 males, median age of 74 years) were enrolled (Table 1). The median HD period was 40 months. The most common primary illness was diabetic nephropathy, observed in 31 patients. Regarding vascular access, arteriovenous fistula, arteriovenous graft, and permanent vascular catheter occurred in 78, 12, and 2 patients, respectively.

**Table 1:** Patient demographics.

Characteristics	Median (IQR) or number
Number of cases, n	92
Age (years)	74 (68–81)
Sex (female/male), n	24/68
Dialysis period (months)	40 (24–99)
Primary illness, n	
Diabetic nephropathy	31
Nephrosclerosis	11
Chronic glomerulonephritis	9
Polycystic kidney disease	4
IgA nephropathy	3
Unknown	26
Others	8
Vascular access, n	
Arteriovenous fistula	78
Arteriovenous graft	12
Permanent vascular catheter	2

*IQR* interquartile range.

### Dialysis setting, examination findings, and complications

Data on a total of 346 HD sessions were obtained and analyzed (Table 2). The median HD time was 4.1 h; there were 81 hemodiafiltration sessions. Regarding anticoagulation agents, unfractionated heparin and low molecular weight heparin were administered in 244 and 102 HD sessions, respectively.

**Table 2:** Dialysis setting and pre-dialysis examination findings.

Characteristics	Median (IQR) or number
Number of dialysis, n	346
Dialysis time (hours)	4.1 (3.6–4.1)
Dialysis type, n	
Hemodialysis	265
Hemodiafiltration	81
Dialysis membrane, n	
Polysulfone	147
Polyethersulfone	118
Polymethyl methacrylate	46
Cellulose triacetate	33
Polyester polymer alloy	2
Medication, n	
Unfractionated heparin	244
Low-molecular-weight heparin	102
Pre-dialysis laboratory data	
Sodium (mEq/L)	139 (137–141)
Potassium (mEq/L)	4.7 (4.2–5.2)
Chlorine (mEq/L)	102 (99–104)
Blood urea nitrogen (mg/dL)	61.0 (52.0–69.9)
Creatinine (mg/dL)	9.52 (7.77–11.18)
Hemoglobin (g/L)	11.2 (10.5–11.9)
Hematocrits (%)	34.7 (32.1–37.0)
Brain natriuretic peptide (pg/mL)	297.7 (165.6–649.4)
Syndecan-1 (ng/mL)	67.7 (39.8–129.9)
Pre-dialysis chest radiography findings	
Cardio-thoracic ratio (%)	51.4 (48.0–55.0)

*IQR*, interquartile range.

At pre-HD, the median serum syndecan-1 concentration was 67.7 ng/mL (interquartile range [IQR] 39.8–129.9), while brain natriuretic peptide (BNP) concentration was 297.7 pg/mL (IQR 165.6–649.4). In addition, the median cardio-thoracic ratio on the chest radiograph was 51.4% (IQR 48.0–55.0) (Table 2). HD complications occurred in 68 sessions; all were intradialytic hypotension, four of which resulted in the discontinuation of HD. No events of arrhythmia or chest pain occurred (Table 3).

**Table 3:** Complications and findings on examination at post-dialysis.

Complications	Number
Total	68
Hypotension	68
Chest pain	0
Arrhythmia	0
D iscontinuation of dialysis due to complications	4
Post-dialysis laboratory data	Median (IQR) or number
Blood urea nitrogen (mg/dL)	19.7 (15.6–24.7)
Creatinine (mg/dL)	3.7 (2.9–4.6)
Atrial natriuretic peptide (pg/mL)	87.6 (55.7–143.8)
Syndecan-1 (ng/mL)	98.3 (50.8–173.4)
Post-dialysis chest radiography findings	
Cardio-thoracic ratio (%)	51.2 (48.2–54.6)
Dialysis efficiency	
Kt/V	1.09 (0.97–1.21)
Volume of water removed/pre-dialysis body weight (%)	4.78 (3.92–5.68)

*IQR*, interquartile range.

At post-HD, the median serum syndecan-1 concentration was 98.3 ng/mL (IQR 50.8–173.4) while atrial natriuretic peptide (ANP) concentration was 87.6 pg/mL (IQR 55.7–143.8). In addition, the median cardio-thoracic ratio on the chest radiograph was 51.2% (IQR: 48.2–54.6), and the median Kt/V-value, an index of dialysis efficiency, was 1.09 (Table 3).

### Relationship between serum syndecan-1 concentration and complications during HD

**Figure 1:**
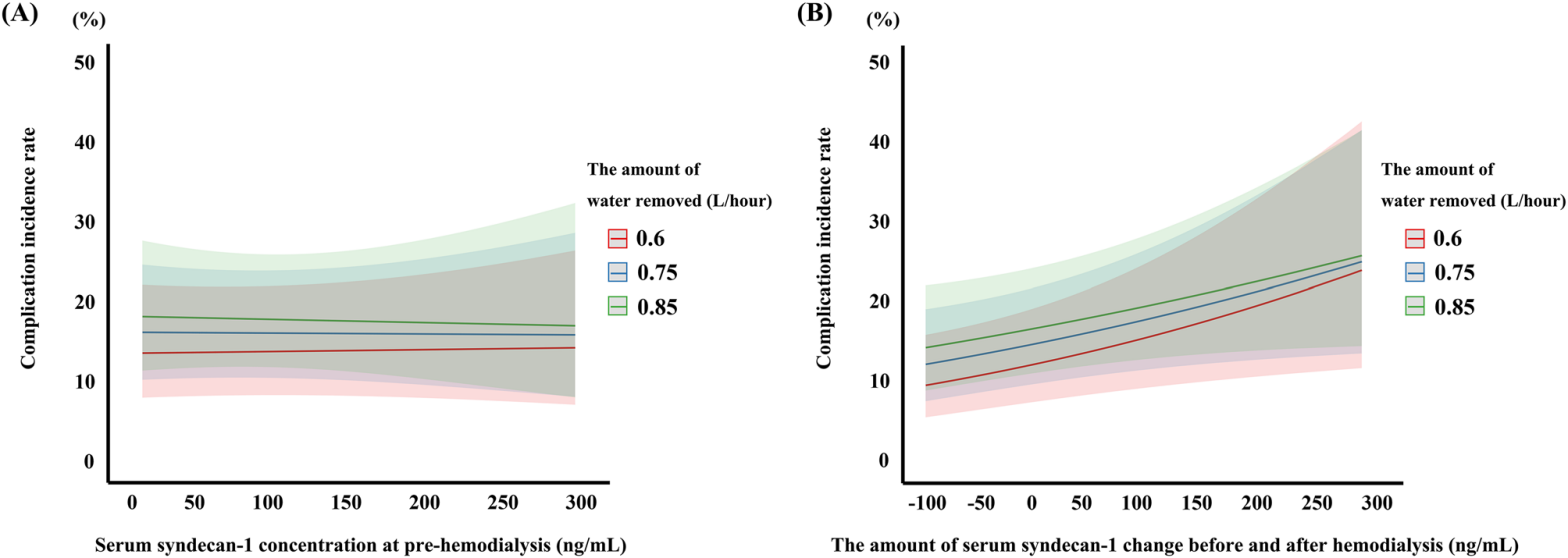
Association between the incidence rate of complications and syndecan-1 levels. Association between complication incidence rates and (**A**) serum syndecan-1 concentration at pre-hemodialysis, (**B**) the amount of serum syndecan-1 change before and after hemodialysis. This figure indicates the predicted value of each parameter over time in patients with the amount of water removed: 0.6 L/hour (red line), 0.75 L/hour (green line), and 0.85 L/hour (blue line). The light-colored area around each solid line indicates the 95% confidence interval.

**Figure 2:**
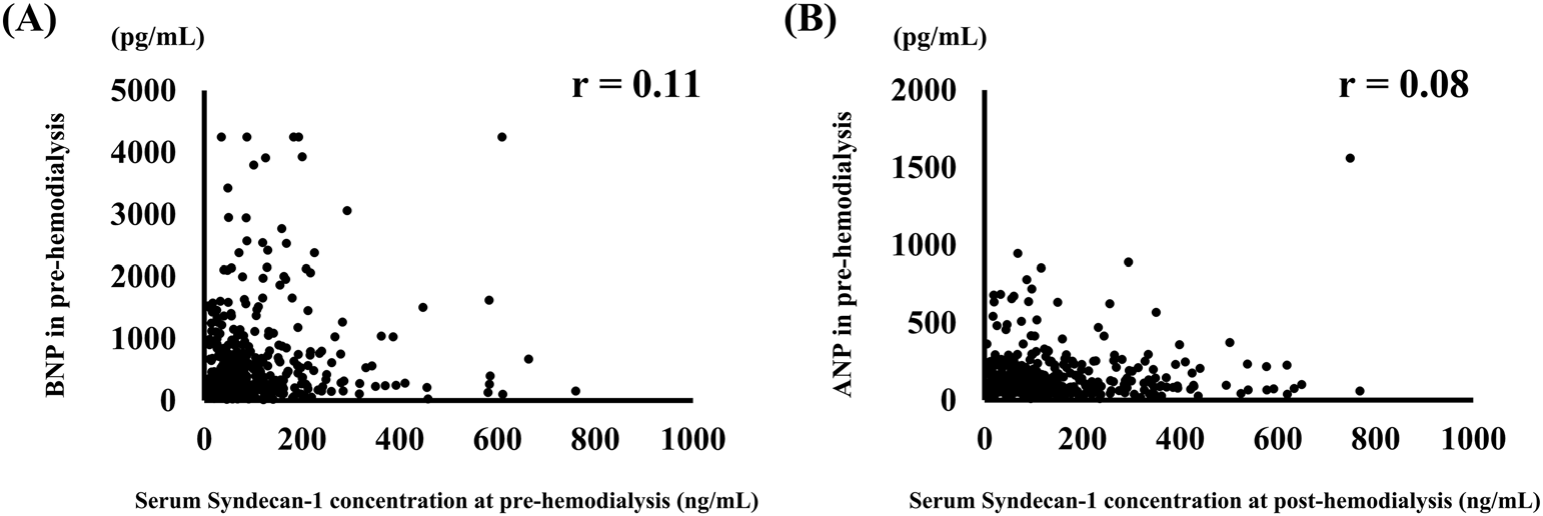
Correlation between serum syndecan-1 levels and brain natriuretic peptide (BNP) or atrial natriuretic peptide (ANP). Correlation between (**A**) BNP and serum syndecan-1 concentration in pre-hemodialysis and (**B**) ANP and serum syndecan-1 concentration at post-hemodialysis. *r* correlation coefficient.

According to the generalized estimating equations (GEE) model adjusted for age, sex, BNP, and amount of water removed per hour, no association was found between serum syndecan-1 concentration at pre-HD and the occurrence of intradialytic hypotension (odds ratio [OR]: 1.001, 95% confidence interval [CI]: [0.993, 1.009], *P* = 0.749, Table 4, Fig. 1A). However, a GEE model using the same adjustment factors showed that the greater the amount of change in serum syndecan-1 concentration before and after HD, the higher the occurrence of complications (OR 1.005, 95% CI [1.001, 1.009], *P* = 0.006, Table 5, Fig. 1B). Similar results were observed when the amount of water removed per hour was corrected for dry weight of patients (see Supplementary Fig. S1 and Supplementary Tables S1 and S2 online). Furthermore, the association between change in serum syndecan-1 concentration before and after HD and complications differed significantly by the amount of water removed per hour (*P* = 0.017). Notably, there was no relationship between the syndecan-1 levels at pre-HD and BNP (Fig. 2A). Likewise, there was no correlation between syndecan-1 concentration at post-HD and ANP concentration (Fig. 2B).

**Table 4:** Relationship between syndecan-1 concentration at pre-hemodialysis and complications during hemodialysis.

Variable	OR	95% LCL	95% UCL	*P*-value
Pre-hemodialysis concentration of syndecan-1	1.001	0.993	1.009	0.749
Amount of water removed/hour	3.944	0.720	21.596	0.114
Age	1.017	0.977	1.059	0.406
Sex	2.222	1.001	4.933	0.050
log BNP	1.006	0.724	1.396	0.973
Interaction between the amount of syndecan-1 change and amount of water removed/hour	0.998	0.986	1.010	0.757

*LCL* lower confidence limit, *UCL* upper confidence limit.

**Table 5:** Relationship between the amount of syndecan-1 change and complications during hemodialysis.

Variable	OR	95% LCL	95% UCL	*P*-value
Amount of syndecan-1 change	1.005	1.001	1.009	0.006
Amount of water removed/hour	4.159	1.233	14.036	0.022
Age	1.016	0.975	1.057	0.455
Sex	2.124	0.993	4.546	0.052
log BNP	1.026	0.739	1.425	0.878
Interaction between the amount of syndecan-1 change and amount of water removed/hour	0.996	0.993	0.999	0.017

*LCL* lower confidence limit, *UCL* upper confidence limit.

## Discussion

The present study revealed that the amount of change in serum syndecan-1, a marker of vascular endothelial glycocalyx damage, before and after HD was associated with the development of intradialytic hypotension. Various mechanisms, one of which may be vascular endothelial glycocalyx, have been postulated for the pathophysiology of intradialytic hypotension.

Intradialytic hypotension has become one of the most common complications of HD in clinical practice^14–17^. In the present report, all complications observed during HD were hypotension. While intradialytic hypotension causes significant patient distress during HD, it is also strongly associated with vascular access failure, cardiovascular events, end organ damage, and mortality, highlighting the need to optimize prevention and treatment strategies^2^.

The decrease in intravascular volume that causes hypotension during HD can be due to a combination of factors. For example, excessive ultrafiltration decreases cardiac output. Cardiac output during HD is also determined primarily by intravascular volume and arterial vascular resistance^18^. In some patients with HD, sympathetic discharge and baroreceptor sensitivity are thought to decrease the regulation of arterial vascular resistance^19,20^. Thus, intradialytic hypotension is thought to decrease blood pressure through a combination of factors that decrease intravascular volume, such as ultrafiltration rate, cardiac output, and arteriolar tone. However, other factors may also decrease intravascular volume.

Recent reports suggest that HD itself may impair the endothelial glycocalyx. It is speculated that HD may increase vascular permeability and decrease intravascular volume^5^. Syndecan-1 is considered a marker of an impaired vascular endothelial glycocalyx^7–9^.

The endothelial glycocalyx is injured by chronic kidney disease and increased plasma volume and is also impaired in HD patients^5,21^. In previous studies, serum syndecan-1 levels were approximately 20 ng/mL in healthy participants^22^, whereas in the present study, the median syndecan-1 level was 67.7 ng/mL (IQR 39.8–129.9) in patients undergoing HD. This result confirms previous studies showing that endothelial glycocalyx is damaged in HD patients. Moreover, the median syndecan-1 concentration was increased post-HD compared with pre-HD, which is consistent with previous reports.

It has also been suggested that syndecan-1 may reflect vascular capacitance load^23^. Conversely, ANP secretion is stimulated by atrial muscle stretch due to atrial pressure, while BNP is secreted when a ventricular load is applied^24^. In this study, there was no correlation between BNP and syndecan-1 or ANP and syndecan-1, suggesting that syndecan-1 may be a different indicator of HD than ANP or BNP.

As such, the results hint at a potential connection between intradialytic hypotension and the dynamics of serum syndecan-1 levels, which may serve as a marker of endothelial injury in HD patients. However, clarifying that this relationship is associative rather than causal is crucial, providing a more accurate reflection of our findings. Further research is warranted to elucidate the underlying mechanisms involved in this intriguing association. No direct treatment for vascular endothelial glycocalyx has yet been established^25^. Since less water removal per hour reduces vascular endothelial glycocalyx damage, slow water removal is one way to prevent intradialytic hypotension. Likewise, using nafamostat mesylate as an anticoagulant during HD reduces glycosylation damage to the vascular endothelium^5^. Therefore, nafamostat mesylate may be a good alternative to unfractionated heparin or low molecular weight heparin in patients with intradialytic hypotension. More detailed studies are required in the future to prove it.

This study had several limitations. First, most patients underwent HD for less than 4 h; therefore, accurate examination of long HD sessions was impossible. Second, the study used fewer anticoagulants and fewer types of dialysis membranes. Third, this study did not measure other biomarkers of glycocalyx damage, such as serum hyaluronic acid and hyaluronidase levels. Other limitations of this study included the small number of patients (92), even though 346 dialysis sessions were analyzed, and the lack of uniformity in HD methods, vascular access, dialyzers, and anticoagulation therapy among the patients included.

Moreover, we only collected blood samples as required in clinical settings but did not obtain post-dialysis hemoglobin data. Thus, we included the amount of water removal during dialysis as an adjustment factor. Hence, we think that changes in syndecan-1 values are not simply due to hemoconcentration. However, this is not direct evidence of a relation between hemoconcentration and changes in syndecan-1.

This study showed that the quantitative assessment of endothelial glycocalyx injury by measuring the concentration of serum syndecan-1 during HD is associated with intradialytic hypotension. This finding, which may be useful for preventing intradialytic hypotension, suggests that impairment of the vascular endothelial glycocalyx may be considered a cause of intradialytic hypotension.

## Methods

### Patients

We enrolled outpatients who underwent HD at the Gifu Seiryu Hospital between April and July 2022. Patients aged < 20 years and those who underwent plasma apheresis, plasma exchange, and double filtration plasma therapy were excluded.

### Ethics approval and consent to participate

The study conformed to the principles outlined in the Declaration of Helsinki^10^. Ethics approval was obtained from the Medical Ethics Committee of the Gifu University Graduate School of Medicine, Gifu, Japan (Approval No.: 2022–167). The need for obtaining informed consent from participants was waived by the medical ethics committee because of the study’s retrospective nature. Before initiation, the study was registered in the UMIN Clinical Trials Registry (Registry Number: UMIN000050656).

### Data collection and study design

This was a single-center, retrospective, observational study conducted at the Gifu Seiryu Hospital, Gifu, Japan. Patients undergoing outpatient maintenance HD at this hospital undergo blood tests at the beginning of each month. Excess serum from these blood tests was used as the blood sample. All laboratory data except serum syndecan-1, dry weight, and other patient attributes were extracted from the hospital’s electronic medical records. Serum syndecan-1 concentrations were measured using an enzyme-linked immunosorbent assay (950.640.192; Diaclone, Besancon, Cedex, France). Data were analyzed retrospectively. As a measure of dialysis efficiency, Kt/V was calculated as previously described^11^.

Complication events, as the primary outcome, were defined as intradialytic hypotension, arrhythmia, and subjective symptoms such as chest pain, nausea, general malaise, and leg cramps during HD. Intradialytic hypotension was defined as a decrease in either systolic blood pressure of 20 mm Hg or mean arterial pressure of 10 mm Hg leading to symptoms, as indicated by the National Kidney Foundation’s Kidney Disease Outcomes Quality Initiative guidelines^12^.

### Statistical analysis

Data for 423 observations were collected from 95 cases based on feasibility. Since the standard deviation (SD) of syndecan-1 was expected to be approximately 100 from previous studies, when the event rate was considered as approximately 20%, the power to detect an OR of 1.5 for a 1 SD increase of syndecan-1 using the logistic regression model was 90.4%. The power to detect an OR of 1.4 was guaranteed by 78%^13^. Since this study involved multiple correlated data (repeated data) from a single patient, more power than the above was expected to be guaranteed.

The GEE model was used to reveal the association between the serum syndecan-1 concentration at pre-HD and hypotension. In the model, we included an interaction term between syndecan-1 and water removed per hour to account for the modifying effect of water removed per hour on the association between syndecan-1 and hypotension. Age, sex, and BNP were included in the model as covariates to adjust for confounders^5^. These variables were selected a priori based on previous studies. A logarithmic transformation was applied since the BNP did not follow a normal distribution. To confirm the association between change in syndecan-1 before and after HD and hypotension, we used a GEE model with change in syndecan-1 before and after HD instead of syndecan-1 in pre-HD in the above model. As the implications of water removed may differ depending on body size, we conducted a supplementary sensitivity analysis using the amount of water removed per hour divided by dry weight of patients as an independent variable. There were no missing values in the data used in the analyses. A two-sided *P* value < 0.05 was considered statistically significant. R version 4.3.1 (R Foundation for Statistical Computing, Vienna, Austria) was used for statistical analyses.

### Supplementary Information

Supplementary Information.

## Data Availability

The raw data supporting the conclusions of this article will be made available from the corresponding author without undue reservation.
