# Supplementary material for: Investigation of the relationship between intradialytic hypotension during hemodialysis and serum syndecan-1 concentration
Source: Sci Rep. 2023 Oct 5;13:16753. doi: 10.1038/s41598-023-44094-7 (PMC10556083; doi:10.1038/s41598-023-44094-7)
Supplement: Supplementary file 1 — Supplementary Information. [file 41598_2023_44094_MOESM1_ESM.docx]

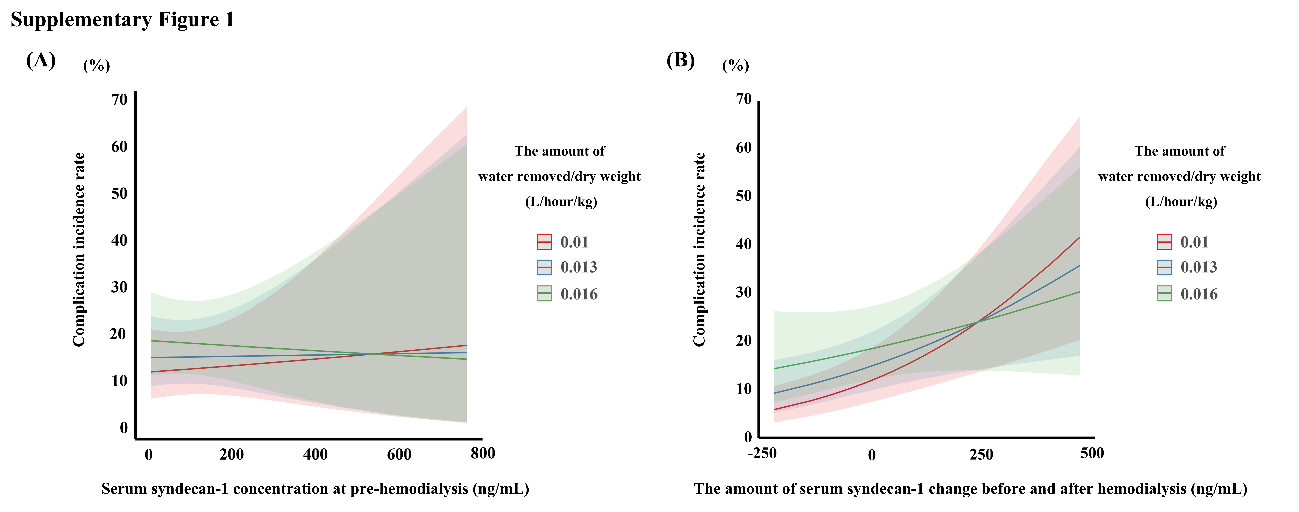


**Supplementary Fig. S1. Association between the incidence rate of complications and syndecan-1 levels.**

Association between complication incidence rates and (A) serum syndecan-1 concentration at pre-hemodialysis, (B) the amount of serum syndecan-1 change before and after hemodialysis. This figure indicates the predicted value of each parameter over time in patients with the amount of water removed corrected by dry weight: 0.01 L/hour/kg (red line), 0.013 L/hour/kg (green line), and 0.016 L/hour/kg (blue line). The light-colored area covering each solid line indicates the 95% confidence interval.

**Supplementary Table S1. Relationship between syndecan-1 concentration at pre-hemodialysis and complications during hemodialysis**

| **Variable** | **OR** | **95% LCL** | **95% UCL** | **P-value** |
| --- | --- | --- | --- | --- |
| Pre-hemodialysis concentration of syndecan-1 | 1.002 | 0.997 | 1.008 | 0.454 |
| Amount of water removed/hour/dry weight/0.001* | 1.085 | 1.000 | 1.178 | 0.050 |
| Age | 1.008 | 0.967 | 1.050 | 0.717 |
| Sex | 1.999 | 0.926 | 4.315 | 0.078 |
| log BNP | 0.979 | 0.690 | 1.388 | 0.905 |
| Interaction between the amount of syndecan-1 change and amount of water removed/hour | 0.858 | 0.619 | 1.190 | 0.359 |

*LCL,* lower confidence limit; *UCL,* upper confidence limit.; *OR*, odds ratio.

*OR and 95% CLs for increments of 0.001 are shown.

**Supplementary Table S2. Relationship between the amount of syndecan-1 change and complications during hemodialysis**

| **Variable** | **OR** | **95% LCL** | **95% UCL** | **P-value** |
| --- | --- | --- | --- | --- |
| Amount of syndecan-1 change | 1.007 | 1.004 | 1.010 | <0.001 |
| Amount of water removed/hour/dry weight/0.001* | 1.089 | 1.017 | 1.166 | 0.014 |
| Age | 1.007 | 0.966 | 1.050 | 0.746 |
| Sex | 1.872 | 0.893 | 3.925 | 0.097 |
| log BNP | 0.996 | 0.702 | 1.411 | 0.980 |
| Interaction between the amount of syndecan-1 change and amount of water removed/hour | 0.702 | 0.544 | 0.905 | 0.006 |

*LCL,* lower confidence limit; *UCL,* upper confidence limit; *OR*, odds ratio.

*OR and 95% CLs for increments of 0.001 are shown.
